# Flexible PZT Thin Film Tactile Sensor for Biomedical Monitoring

**DOI:** 10.3390/s130505478

**Published:** 2013-04-25

**Authors:** Hong-Jie Tseng, Wei-Cheng Tian, Wen-Jong Wu

**Affiliations:** 1 Department of Engineering Science and Ocean Engineering, National Taiwan University, Taipei 10617, Taiwan; E-Mail: hjtseng@mems.iam.ntu.edu.tw; 2 Department of Electrical Engineering, National Taiwan University, Taipei 10617, Taiwan; 3 Graduate Institute of Electronics Engineering, National Taiwan University, Taipei 10617, Taiwan; 4 Graduate Institute of Biomedical Electronics and Bioinformatics, National Taiwan University, Taipei 10617, Taiwan

**Keywords:** PZT thin-film, sol-gel, sensor, biomedical

## Abstract

This paper presents the development of tactile sensors using the sol-gel process to deposit a PZT thin-film from 250 nm to 1 μm on a flexible stainless steel substrate. The PZT thin-film tactile sensor can be used to measure human pulses from several areas, including carotid, brachial, finger, ankle, radial artery, and the apical region. Flexible PZT tactile sensors can overcome the diverse topology of various human regions and sense the corresponding signals from human bodies. The measured arterial pulse waveform can be used to diagnose hypertension and cardiac failure in patients. The proposed sensors have several advantages, such as flexibility, reliability, high strain, low cost, simple fabrication, and low temperature processing. The PZT thin-film deposition process includes a pyrolysis process at 150 °C/500 °C for 10/5 min, followed by an annealing process at 650 °C for 10 min. Finally, the consistent pulse wave velocity (PWV) was demonstrated based on human pulse measurements from apical to radial, brachial to radial, and radial to ankle. It is characterized that the sensitivity of our PZT-based tactile sensor was approximately 0.798 mV/g.

## 1. Introduction

Capacitive-based, piezoresistive-based, and piezoelectric-based sensors are commonly used for tactile sensing. A flexible membrane and gap are typically included in a capacitive-based sensor, which can be widely used for applications in mobile robot contact force arrays [1], pressure sensors [2,3], proximity sensors [4], and tactile sensing arrays [5]. The applied pressure of piezoresistive-based tactile sensors [6] results in resistance changes and can be used for force sensors [7], pressure sensors [8], and tactile sensors [9]. However, capacitive-based tactile sensors typically require high voltage operation, whereas piezoresistive-based tactile sensors encounter signal drift caused by temperature changes. Therefore, piezoelectric-based sensors were chosen to examine tactile sensing applications.

Among various piezoelectric-based materials, lead zirconium titanate (PZT) thin-film is an excellent ferroelectric material for tactile sensor applications. PZT-based sensors have several advantages, such as high sensitivity, wide frequency bandwidth, and fast response. Thus, these sensors can be widely used for micro-electromechanical systems (MEMS) applications, such as in the areas of transducers [10], micromirrors [11,12], switches [13], gas sensors [14], pyroelectric sensors [15,16], energy harvesting devices [17–19], and tactile sensors [20]. Tactile sensors fabricated using MEMS offer the advantages of small size, mature technologies, and low cost processing. PZT thin-films fabricated using the sol-gel method provides the advantages of easy processing, low annealing temperature, and excellent piezoelectric characteristics. Accurate Zr/Ti element composition can be controlled in the sol-gel deposition process; thus, a composite material with a high ferroelectric property (Zr/Ti with a ratio of 52:48) can be obtained.

Various sensors, such as piezoelectric-based sensors [21–25], optical sensors [26–29], and laser Doppler [30] sensors have been used for measurements of human body pulses at various artery regions. For piezoelectric-based tactile sensors, the mechanical energy can be transferred to electrical energy using an applied pressure, and the sensors have the advantages of high sensitivity, improved hysteresis, excellent repeatability, and high durability. In addition, flexible materials, such as aluminium nitride (AlN), lead-lanthanum-zirconate-titanate (PLZT), and polyvinylidene difluoride (PVDF) can be used for piezoelectric-based sensing applications.

We developed a novel sol-gel process to fabricate the PZT thin-film on a flexible stainless steel substrate. The proposed process has the advantages of simple fabrication with a lower cost for various applications. The fabricated tactile sensors have high sensitivity and fast strain response, and the flexible substrate enabled rugged human body monitoring, such as sensing human pulses at various topologies of several human artery areas. For example, the topology of the carotid artery area is flat, whereas the finger is curved. The PZT-based tactile sensors fabricated using the novel sol-gel process on a flexible stainless steel substrate can be used as rugged human pulse sensors with high performance. Future work application, the PZT thin film tactile sensor on stainless steel substrate and can be applied the structure health monitoring such as the bridge [31] or engineering structure [32]. In addition, the flexible PZT tactile sensor to apply energy harvesting [33], large area tactile sensors array [34], robot hard [5], touch sensor [35], pressure sensor [36] and fingerprint [37].

## 2. Experimental Section

### 2.1. Solution Based Process for PZT Thin Film Formation

During the sol-gel deposition process, the PZT solution was spun on the stainless steel substrate. Two chemical reactions were performed during the sol-gel process, including hydrolysis and condensation reactions. The chemical reaction equations [38] of the hydrolysis reactions are shown in Equation (1), whereas the chemical reaction equations of the condensation reactions are shown in Equations (2) and (3):

(1) $$\text{M}(\text{OR})+{\text{H}}_{2}\text{O}\to \text{M}(\text{OH})+\text{ROH}$$

(2) $$\text{M}(\text{OH})+\text{M}(\text{OH})\to \text{M}-\text{O}-\text{M}+{\text{H}}_{2}\text{O}$$

(3) M(OH)+M(OR)→M−O−M+ROH

The PZT solution used to prepare the PZT thin-film in the sol-gel process consisted of lead acetate, zirconium n-prop-oxide, and titanium iso-propoxide, and the molar ratio of these compounds was 1.1:0.52:0.48. Organic solvents, such as acetic acid, lactic acid, glycerol, and ethylene glycol can be added to the mixed powders to prepare the PZT mixing solution.

**Figure 1.**
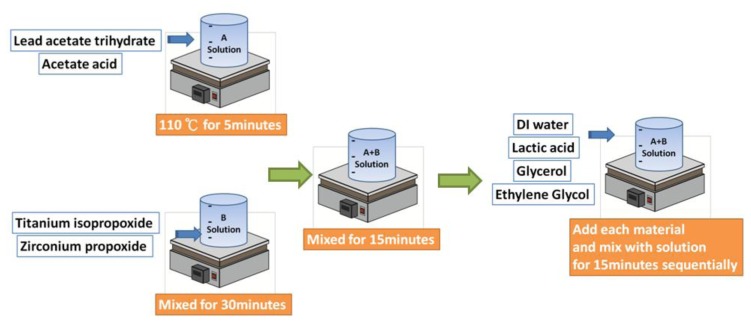
Preparation process of PZT solution Device.

The preparation steps of the PZT mixing solution are shown in Figure 1. First, Solution A was prepared by mixing the zirconium n-prop-oxide and titanium iso-propoxideat at room temperature for 30 min. Second, Solution B was prepared by mixing lead acetate and acetic acid at 110 °C for 5 min. Next, Solution A was mixed with Solution B for 15 min. The mixture of Solutions A and B was subsequently mixed with DI water for 15 min, followed by mixing with lactic acid for 15 min and glycerol/ethylene glycol for 15 min to obtain the final solution with a concentration of 1. 9 M.

### 2.2. Device Fabrication

**Figure 2.**
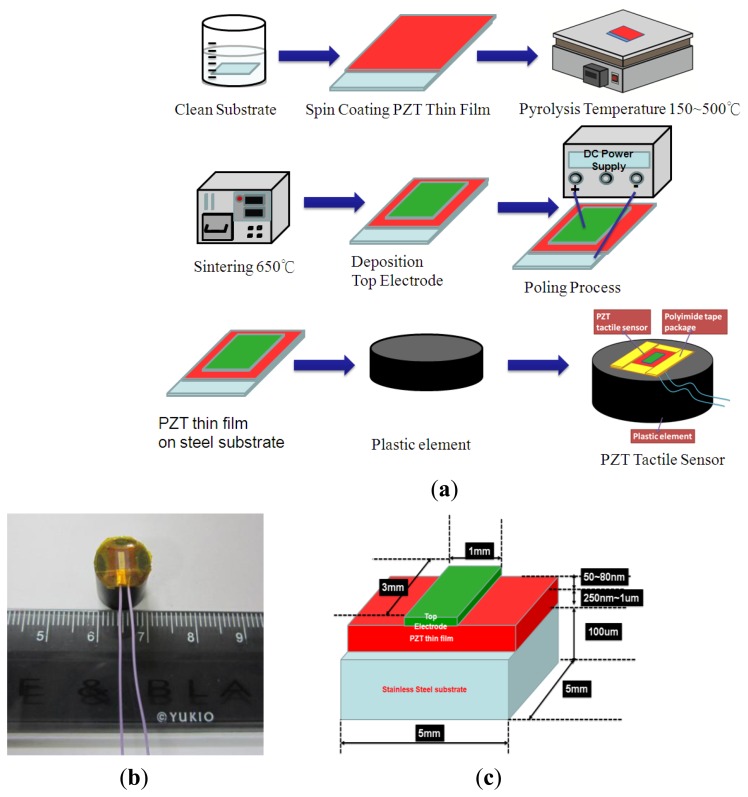
(**a**) Fabrication process of PZT flexible tactile sensors, (**b**) a packaged PZT thin film tactile sensor, (**c**) detailed geometries and dimensions of the different parts of tactile sensor.

The 100 μm-thick stainless steel substrate for the tactile sensor was cleaned using ultrasound in acetone, isopropyl alcohol, and DI water for 10 min. Next, the PZT sol-gel was spin-coated on the stainless steel substrate followed by the pyrolysis processes at temperatures of 150 °C and 500 °C for 10 min and 5 min, respectively. After the pyrolysis process, the thin-film was annealed at 650 °C for 10 min. Finally, the Au, which served as the top electrode, was deposited using a shadow mask and a metal sputter on the PZT thin-film to complete the sensor fabrication process. A poling voltage of approximately 50 V was applied to the PZT film to activate the PZT tactile sensor. Finally, the polyimide tape was used to cover the sides and corners of the PZT thin film sensor and to secure the sensor on the plastic element. The overall fabrication and packaging process were shown in Figure 2(a). The packaged sensor was shown in Figure 2(b). The detailed geometries and dimensions of the different parts of tactile sensor were show in Figure 2(c).

### 2.3. Tactile Sensors Experimental Set-Up

**Figure 3.**
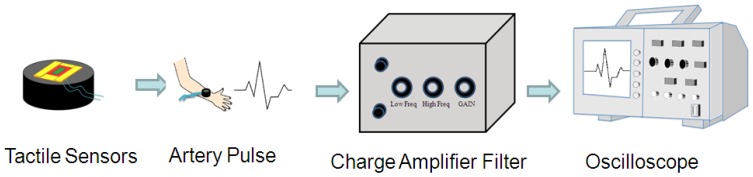
Experimental set-up for PZT flexible tactile sensor characterizations.

As shown in Figure 3, the PZT-based tactile sensors were used to measure human pulses. The signals from the sensor were conditioned using the self-built testing system, including a charge amplifier (Measurement Specialties, piezo film lab amplifier, Hampton, VA, USA) and an oscilloscope (Agilent technology, Digital oscilloscope Agilent Infiniium DSO9404A, Santa Clara, CA, USA). Various regions from the human body were chosen for pulse measurements, such as the carotid artery, the brachial artery, the finger, the ankle artery, and heartbeats. The tactile sensor was connected to the charge amplifier to magnify the sensor signal, and a filter was used to remove the undesirable signals outside the targeted frequency (1–10 Hz). Finally, the conditioned pulse signals were observed and recorded using an oscilloscope.

## 3. Results and Discussion

### 3.1. Characterizations of PZT Films and Tactile Sensors

#### 3.1.1. Surface Morphology and Crystal Orientation of PZT Thin Films

**Figure 4.**
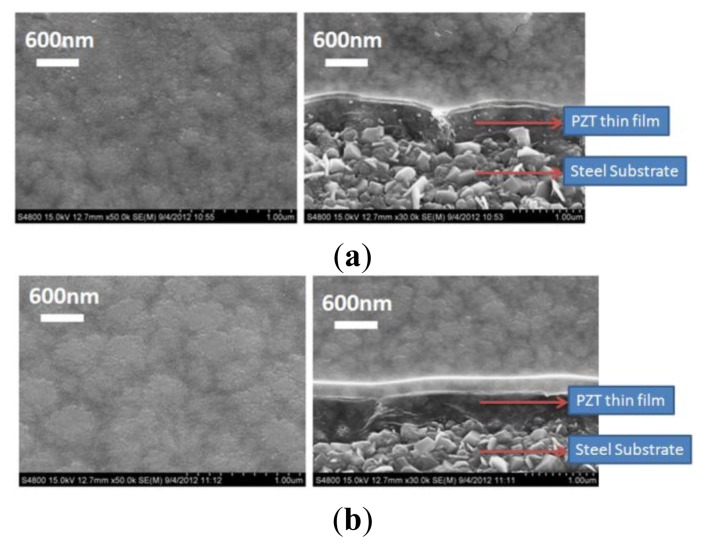

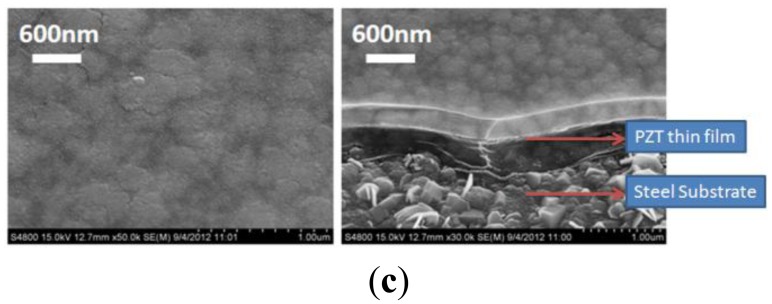
PZT thin films deposited from single or multilayer spin coating (**a**) single layer (**b**) double layer (**c**) triple layer.

**Figure 5.**
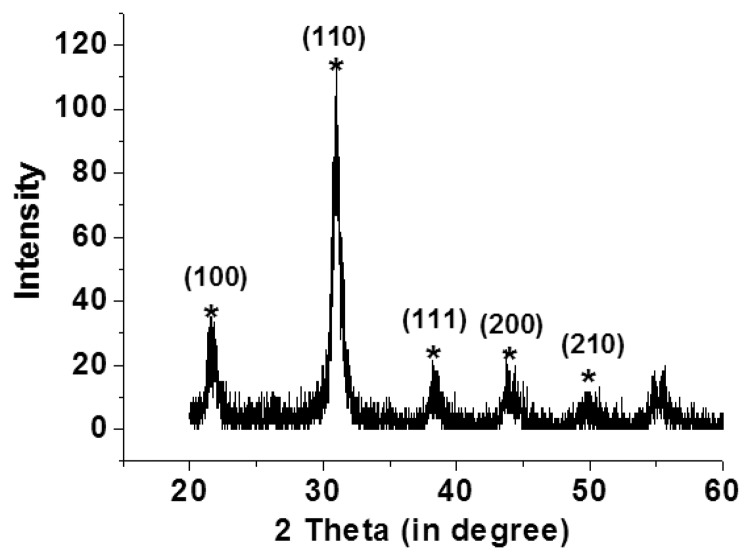
A PZT thin film crystal orientation on flexible stainless steel substrate.

Scanning electron microscopy (Hitachi, S4800, Tokyo, Japan) and an X-ray diffraction system (BRUKER, D8-SSS, Berlin, Germany) were used to characterize the fabrication results. Single and multi-layer deposition of the PZT thin-film on the flexible stainless steel substrate using the sol-gel process are shown in Figure 4(a–c). A PZT thin-film layer of 300 nm was obtained using a single spin-coating process. A 900 nm layer was obtained by spin coating the PZT thin-film three times. Uniform grains were clearly observed on the surface of the PZT thin-film on the flexible stainless steel substrate. Various major peaks at the main directions of the perovskite phase at <110>, <100>, and <111> were observed using X-ray diffraction to measure the crystal orientation of the PZT thin film; the PZT thin-film was polycrystalline, as shown in Figure 5.

#### 3.1.2. Effect of Piezoelectric Material Flexibility on Electrical Performances of Tactile Sensors

Our sensor consists of a piezoelectric material and a stainless steel substrate. Therefore the sensor flexibility is dominated by the properties of the stainless steel substrate material, as the Young's modulus of stainless steel is much higher than the PZT or P(VDF-TrFE) material, as shown in Table 1. Silicon wafers have been commonly used for the sensor substrate but the low Poisson's ratio limits the substrate flexibility.

**Figure 6.**
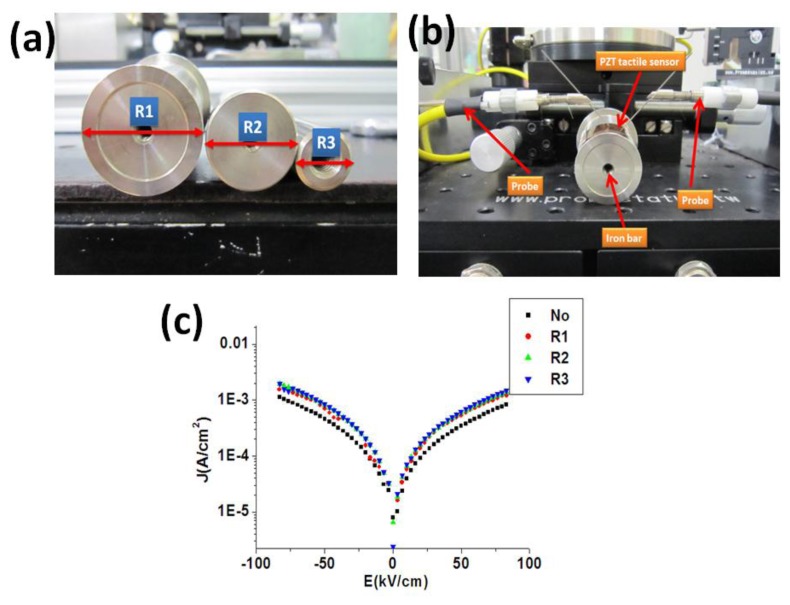
(**a**) Iron bars with different radius, (**b**) test set-up of our sensor flexibility testing, (**c**) J-E curves of our PZT sensor mounted without and with iron bars at different radius of R1–R3.

To study the flexibility of the piezoelectric materials, it was reported that the electrical properties (such as J-E curve) of the flexible sensors under different bending were compared [39,40]. To compare the electrical properties of our piezoelectric material on the stainless steel substrate under various bending situations, we first performed the spin coating of the PZT material on the stainless steel substrate. As show in Figure 6(a,b), the PZT tactile sensor was fixed on the iron bars with different radius followed by the measurement of the material J-E curve of the sensor. From the measurement results, as shown in Figure 6(c), the material J-E curves of our PZT sensor secured at different iron bars showed only minor differences. These bending experiments created more curvature of the sensor than the real situations of the human pulse measurement. Therefore, it was demonstrated that our sensor can be utilized for human body measurement with various topologies by using our PZT material on the stainless steel substrate.

### 3.2. Pulse Measurements on Various Human Body Regions and the Packaged PZT Thin Film Tactile Sensor on Stainless Steel Dangerous Testing

#### 3.2.1. Monitoring of Human Body Pulse Waves

For the human pulse measurement, it is important to use the flexible substrate to accommodate the various surface topologies of the human body. The flexible stainless steel substrate was selected as it was compatible with the fabrication of our targeted piezoelectric material and was more robust than other type of flexible (such as Al foil or polyimide). We also demonstrated a more diverse measurement (six arterial regions of the human body) than other research groups as our senor was able to detect most arterial regions of the human body weather it was close or far away from the human skin surface. The comparisons of our sensor and others were summarized in Table 2.

**Figure 7.**
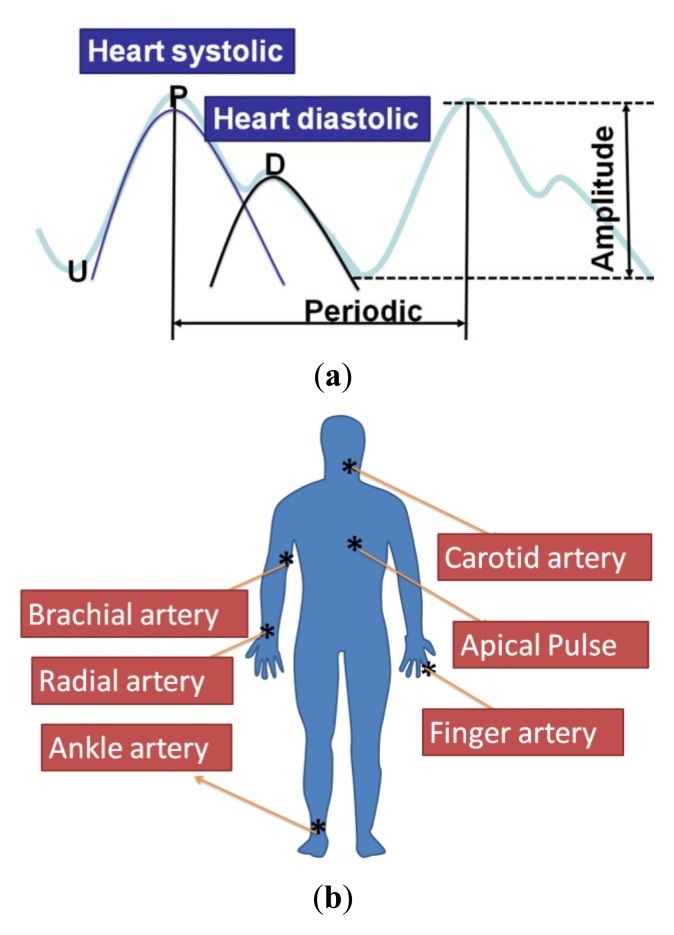
(**a**)The heart systolic to diastolic and to produce the general human pulse waveform (**b**) the PZT tactile sensor to measure the human body artery pulse region.

As shown in Figure 7(a), the general human body pulse waveform (thick aqua line) has two peaks by a human heart: the first peak (P position in the blue line waveform) from the heart systolic pressure and the second peak (D position in the black line) from the diastolic pressure. The period and magnitude of the human body pulse waveform are constant under a resting condition. These repetitive signals were measured using the proposed tactile sensors at various regions of the human body, including the carotid artery, the brachial artery, the radial artery, the finger, the ankle artery, and the apical region, as show in Figure 7(b).

**Figure 8.**
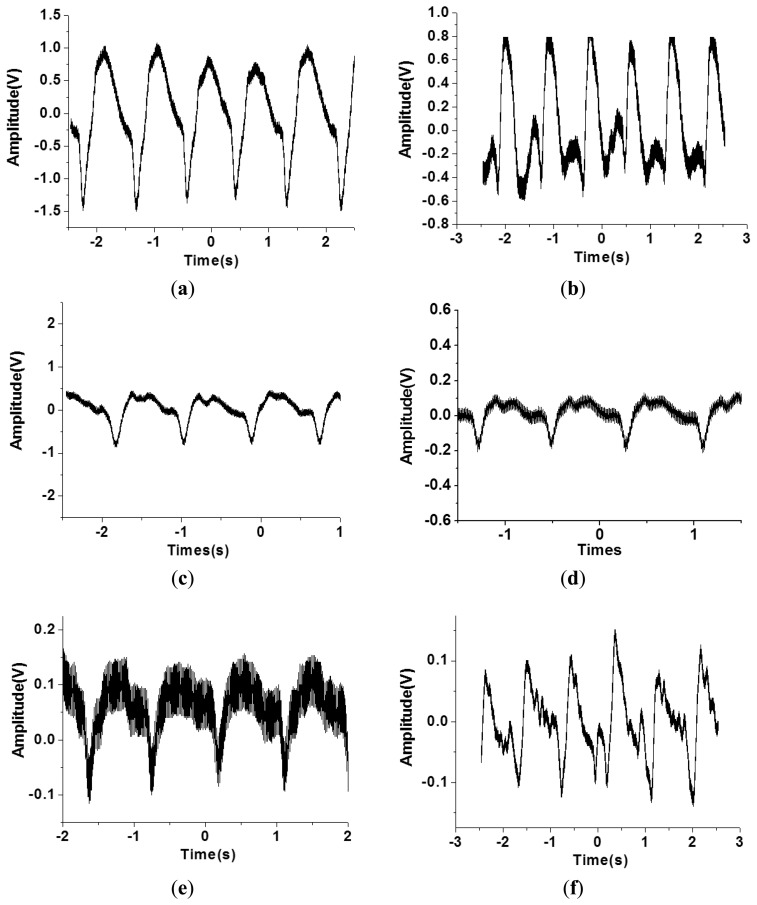
A PZT flexible tactile sensor to sense different human body region (**a**) carotid artery (**b**) apical pulse (**c**) brachial artery (**d**) radical artery (**e**) finger artery (**f**) ankle artery region.

**Figure 9.**
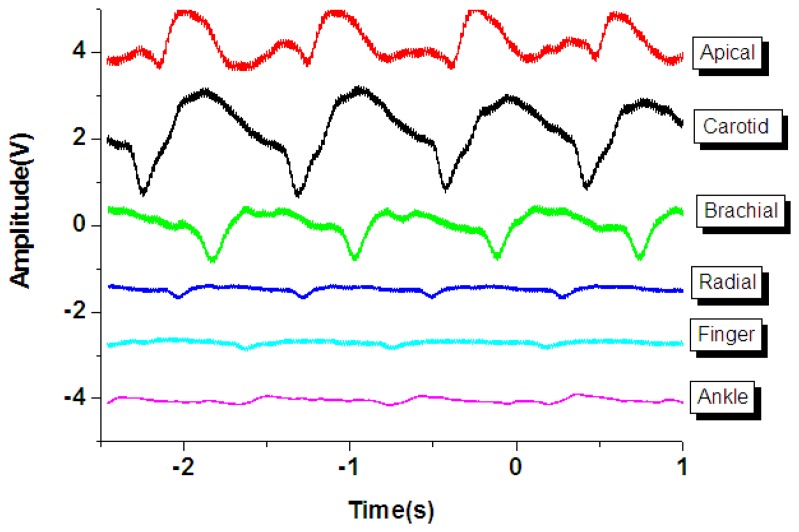
Comparison of pulse amplitude and waveform measured from numerous regions of the human body with our sensor.

However, the geometry and shape of the human body is typically irregular and complex. For example, the topology of the carotid region is smoother compared to that of the finger region. In addition, the amplitude of the pulse signal is highly dependent on the distance between the surface of the skin and the artery underneath the skin. Therefore, the flexible tactile sensor was designed to be adapted to various human body regions and sense various amplitudes of pulses. As shown in Figure 8, the PZT tactile sensors sense the pulses of various human body regions from the carotid artery close to the human head to the ankle artery in the feet. Pulses from the carotid artery were first measured, and a large signal was obtained because of the smooth surface of the detected region. Next, the apical pulse from the heart was sensed for the heartbeat, and the waveform differed from that of the pulse monitored from other regions. In addition, weaker pulses from the finger and radial artery regions were observed compared to the stronger signals in the brachial artery region. However, in the ankle region, the pulse magnitude was large because the ankle artery is close to the surface of the skin. Using the proposed PZT-based flexible tactile sensors, various amplitudes and waveforms can be successfully detected at various areas of the human body, as shown in Figure 9.

#### 3.2.2. Estimate of Human Body Pulse Wave Velocity (PWV)

**Figure 10.**
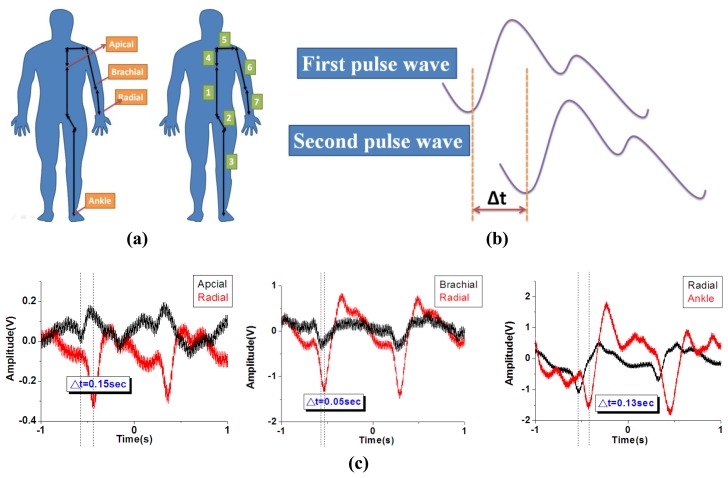
(**a**) PWV definition of the distance from apical to radial, brachial to radial and radial to ankle distance (**b**) The human body PWV calculation (**c**) the time interval between the apical to radial, brachial to radial and radial to ankle.

For the pulse wave velocity (PWV) measurement, the definition of the distance from apical to radial, brachial to radial and radial to ankle distance was shown in Figure 10(a) [47]. The distance from apical to radial artery is defined as “Distance = 4 + 5 + 6 + 7”, the distance from brachial to radial artery is defined as “Distance = 7”, and the distance from radial to ankle artery is defined as “Distance = 1 + 2 + 3 – 4 – 5 – 6 – 7”. The distance was measured with a standard ruler and the actual distance will be varied person by person. Therefore, when performing a real clinical test for a patient, it is important to utilize the correct distance in between each test spot to get accurate measurement. The human body PWV is a crucial index for patient health. As shown in Figure 10(b), the human body PWV can be estimated based on the time difference Δ**t** of the two successive peaks of systolic pressure and diastolic pressure. We synchronized two measurements from differing regions of the human body and recorded the signals, as shown in Figure 10(c). The difference of the path length from the first region to the heart and the other region to the heart was D. Thus, the human body PWV was estimated based on the following formula:

(4) D/Δt

As shown in Table 2, the path distance to the heart between the apical artery and the radial artery is approximately 0.85 m, and the time interval between the heart and radial artery is approximately 0.15 s; therefore, the estimate of the PWV from the apical artery region to the radial artery region is 5.66 m/s. The same methodology can be applied to estimate the pulse wave velocities at various regions, as shown in Table 3.

#### 3.2.3. The Safety Tests of Packaged PZT Thin Film Tactile Sensor on Stainless Steel Substrate

**Figure 11.**
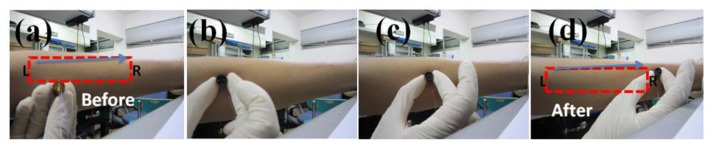
(**a**) The packaged PZT thin film sensor and the area to be touched, (**b**) the packaged tactile sensor touched the human skin, (**c**) the packaged tactile sensor was moved from left to right, (**d**) no scratches were found on the areas the sensor touched.

As shown in Figure 11(a–d), the packaged PZT thin film tactile sensor was touched and moved back and forth on the human skin. During the multiple tests (>100 times), no damages or scratches were found on the human skin. We also compared the safety of the unpackaged stainless steel plate and our packaged PZT tactile sensor. A scratch line was observed on a paper when touching and moving the unpackaged stainless steel plate over a paper while no scratches were observed when using our packaged PZT tactile sensor. From these safety tests, it is concluded that our packaged PZT thin film sensor with a stainless steel substrate is very safe for human pulse measurements.

## 4. Conclusions

We have successfully fabricated PZT flexible tactile sensors using a sol-gel deposition process. The PZT thin-film was deposited on the flexible stainless steel substrate to enable broad pulse detection capability for various human body regions, such as the carotid region, the brachial region, the finger, the ankle artery, the radial artery, and the apical region. The PZT flexible tactile sensor is sensitive to several human body regions with various geometries. The amplitude of the human pulse waveform is large on the carotid artery, whereas that the finger region is small. Using the proposed PZT-based flexible tactile sensors, a wide range of pulse sensing from numerous regions of the human body can be achieved for the monitoring human health. Finally, human body pulse wave velocities from the apical pulse region to the carotid region, the brachial artery region, the finger, and the ankle artery of the human body were estimated and the results were consistent.

## Figures and Tables

**Table 1.** Material properties of PZT, PVDF, silicon and stainless steel.

	**Young's Modulus (GPa)**	**Poisson's Ratio**	**Density (kg/m^3^)**
PZT [41]	70	0.3	7,500
PVDF [42,43]	2∼4	0.34	1.78
Silicon [44]	168.9	0.182	2,330
Stainless steel [41]	193	0.318	8,000

**Table 2.** Comparison of piezo-electric tactile sensors with different flexible substrates.

	**Substrate**	**Application**
PVDF [23]	NA	Radial
PVDF [25]	NA	Brachial, Radial
AlN [21]	Polyimide (8.5 μm)	Finger
AlN [22]	Aluminum foils (1 μm, 16 μm)	Femoral
PZ [45]	NA	Rubber tube
PZ [46]	NA	Elastic tube
PZT (this work)	Stainless steel	Carotid, Brachial, Finger, Ankle, Radial, Apical

**Table 3.** The apical to radial, brachial to radial and radial to ankle human body (PWV).

	**Distance (m)**	**Δt (s)**	**PWV (m/s)**
apical to radial	0.85	0.15	5.66
brachial to radial	0.2	0.05	4
radial to ankle	0.8	0.13	6.15
